# Review of RF Device Behavior Model: Measurement Techniques, Applications, and Challenges

**DOI:** 10.3390/mi15010046

**Published:** 2023-12-25

**Authors:** Haode Li, Jiangtao Su, Ruijin Wang, Zhenyu Liu, Mengmeng Xu

**Affiliations:** 1 School of Electronic Information, Hangzhou Dianzi University, Hangzhou 310018, China; haodeli@alu.hdu.edu.cn (H.L.); 211040064@hdu.edu.cn (R.W.); 221040016@hdu.edu.cn (Z.L.); 212040216@hdu.edu.cn (M.X.); 2 Zhejiang Key Laboratory of Large-Scale Integrated Circuit Design, Hangzhou Dianzi University, Hangzhou 310018, China

**Keywords:** behavior model, nonlinear distortion, parameter extraction, memory effects

## Abstract

This review presents a concise overview of RF (radio frequency) power transistor behavior models, which is crucial for optimizing RF performance in high-frequency applications like wireless communication, radar, and satellites. The paper highlights the significance of accurate modeling in understanding transistor behavior and traces the evolution of behavior modeling techniques. Different behavior modeling strategies, such as LUT (look-up table) based models, polynomial equation-based models, and machine learning based models, are discussed along with their unique characteristics and modeling challenges. The review explores the difference between behavior models and the conventional empirical or physics-based modeling approaches, addressing the challenges of the accurate characterization of transistors at high frequencies and power levels. This paper concludes with an outlook of emerging trends, such as physical models combined with behavior models, shaping the future of RF power transistor modeling for more efficient communication systems.

## 1. Introduction

With the continuous evolution of modern wireless communication technology and the advent of new semiconductor processes and materials, various radio transmission systems are becoming more and more complex, and the application fields are also expanding. In this context, RF front-end circuits and devices, which are crucial parts of communication circuits, have garnered extensive research and attention.

In the process of circuit design, the use of computer-aided design (CAD) software has become a key step in simulation and analysis. These tools can provide valuable information before the actual manufacturing of the circuit. This process significantly reduces the design cost and shortens the design cycle. However, to achieve this goal, an accurate circuit model must be available because only an effective model can provide useful guidance.

For a long time, the modeling methods in the microwave field have mainly focused on two directions: physical basis models and empirical basis models [1,2]. The former requires an in-depth study of the materials, structural parameters and process parameters used in transistor devices to obtain the voltage–current characteristics of the devices. However, as the physical phenomena are relatively difficult to characterize, the physical model in actual circuit design is relatively limited. The latter approach fits the device characteristic curve through an empirical function [3], which improves computational efficiency at the expense of discarding the physical meaning of the parameters. But this also means that the empirical basis model has lost some guiding ability in device design.

Both the physical basis models and empirical basis models require accurate measurement of S-parameters. However, the actual electromagnetic environment is very complex, and the input signal and the output signal usually do not follow a simple linear relationship. Therefore, the limitations of the traditional S-parameter model are particularly prominent with the continuous improvement of the performance requirements of communication systems, where devices are high-power and high-efficiency with a nonlinear output in saturated working areas. Under this working condition of large signal excitation, RF devices will exhibit nonlinear phenomena such as gain compression, harmonic distortion, intermodulation distortion, the self-heating effect, and the memory effect. The physics-based model and the empirical-based model need to provide more parameters to describe these phenomena, which makes the model more complex and the parameter extraction more difficult.

Behavioral modeling has become a research direction that has attracted a lot of attention in recent years [4]. The behavior model is a black box model, and its extraction process does not need to understand the internal structure or equivalent circuit of the device. It only relies on measuring the input and output signals of the black box, and selects the appropriate model structure through appropriate methods and identifies the model parameters according to the port information, thereby establishing a model equivalent to the device characteristics. Since there is no need to understand the physical relationship between the elements inside the device, the behavior model helps to protect intellectual property rights and prevent reverse engineering. Compared with physical models and empirical models, behavioral models have the advantages of low time cost, high modeling efficiency, and sufficient accuracy, and have been widely used in RF front-end circuit and device modeling. This fast and efficient modeling method helps to meet the growing demand for nonlinear system modeling, and promotes its research and application in the field of RF engineering.

**Figure 1:**
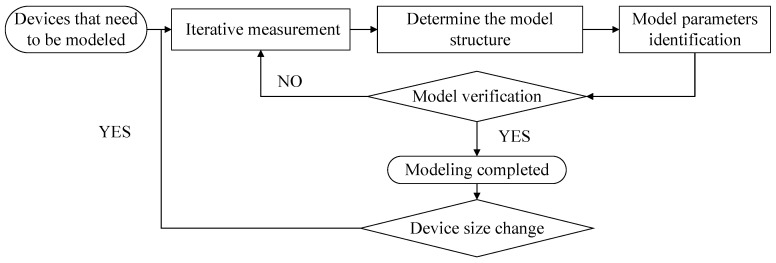
Flow chart of behavior model establishment.

The modeling process of the behavior model is shown in Figure 1 below. This article will introduce each step and emphasize the significant characteristics of the behavior model relative to other types of models.

This paper aims to discuss the latest developments in the progress of RF device behavior models, as most of these methods have their own uniqueness. Starting from the development history, a deep study of different behavior models’ applicable conditions and limitations will be carried out. Since measurement techniques are the precondition to obtaining the necessary data to establish an accurate behavior model, they will be discussed in this paper, as well as the various application fields of the behavior model. These range from the optimization of PA performance, accurate signal integrity analysis, and the modeling of complex electromagnetic interference (EMI) problems, highlighting its importance in the real world. Finally, the challenges faced by behavior models are comprehensively reviewed, with particular attention paid to the high dependence on large-scale test data, and the prospects for future development are pointed out, providing useful insights for research and practice in this field.

## 2. Polynomial Based Behavior Model

The polynomial-based behavior model can be seen as the nonlinear extension of S-parameters. Many researchers have since proposed various behavior models through different modeling strategies [5], although various limitations have existed. This section aims to review the development of these models to help future researchers better select nonlinear behavior models suitable for different applications.

### 2.1. Hot-S Parameters

In the study of linear circuits, the traditional S-parameter has been dominant and has had an important impact on the microwave field for decades. Therefore, when RF engineers began to study the modeling of nonlinear devices, they hoped to extend the concept of S-parameters to the nonlinear field [6,7].

Based on this concept, the Hot-S parameter is introduced [8]. Different from the traditional single excitation source measured conditions of S-parameters, Hot-S parameters introduce the setting of double excitation sources on this basis [9]. It adds a large signal ( $f_{c}$ ) to the input of the device to enable the device to enter the working state, and then measures the response of the small signal ( $f_{s}$ ). In this case, the concept of *a* wave and *b* wave is similar to the classical *S*-parameter:

(1) $$\left[\begin{array}{c} b_{1}\left(f_{s}\right)\\ b_{2}\left(f_{s}\right) \end{array}\right]=\left[\begin{array}{cc} {hotS}_{11} & {hotS}_{12}\\ {hotS}_{21} & {hotS}_{22} \end{array}\right]\left[\begin{array}{c} a_{1}\left(f_{s}\right)\\ a_{2}\left(f_{s}\right). \end{array}\right]$$

In the microwave frequency band, it is usually difficult to directly measure the voltage and current waveforms. Therefore, in the study of the frequency domain behavior model, the traveling wave formula is often used to describe it, which involves the linear combination of port voltage and current. In general, we use $A_{pm}$ to represent the incident wave and $B_{pm}$ to represent the scattered wave.

However, this simple expansion method only considers the influence of the large signal input $f_{c}$ , and does not fully consider the influence of the intermodulation product of the large signal $f_{c}$ and the small signal $f_{s}$ on the model. In order to improve the model, an additional small signal with the same frequency as the driving large signal is introduced and applied at the other end of the device. Since the power of the input small signal is much smaller than that of the driving large signal, we can regard this small signal as a disturbance signal without changing the working state of the device under the condition of ignoring the high-order harmonic response of each port and retaining only the fundamental response. At this time, the Hot-S parameter can be described as:

(2) $$\left[\begin{array}{c} b_{1}\left(f_{s}\right)\\ b_{2}\left(f_{s}\right) \end{array}\right]=\left[\begin{array}{cc} hotS_{11} & hotS_{12}\\ hotS_{21} & {hot}_{22} \end{array}\right]\times \left[\begin{array}{c} a_{1}\left(f_{c}\right)\\ a_{2}\left(f_{c}\right) \end{array}\right]+\left[\begin{array}{c} T_{12}\\ T_{22} \end{array}\right]e^{j2\varphi \left(a_{1}\left(f_{c}\right)\right)}conj\left(a_{2}\left(f_{c}\right)\right).$$

In the above equation, by introducing $T_{12}$ and $T_{22}$ terms, the nonlinear response of the disturbance signal and its conjugate term near the working point of the large signal is described, and the nonlinear behavior is linearized to a certain extent. However, it should be noted that, since the equation only considers the fundamental response, it cannot fully express the influence of nonlinear products.

### 2.2. Poly-Harmonic Distortion Model

As a further improvement of the extended thermal S-parameters, Verspecht et al. proposed a new large-signal scattering parameter technique, which is called the poly-harmonic distortion (PHD) model [10]. The PHD model is derived by strict mathematical derivation based on the description function.

Since the description function represents a time-invariant system, if the incident wave has a certain delay, the scattered wave will also have the same delay. This characteristic is shown as a linear phase shift in the frequency domain. In the description function, the phase of the fundamental excitation signal is used as a reference, and the phase operator *P* is introduced, where $P=e^{j\varphi A_{11}}$ . The phase and amplitude can be separated, thus we can get Formula (3):

(3) $$\begin{array}{c} B_{pm}=F_{pm}\left(\left|A_{11}\right|,A_{12}P^{-2},A_{13}P^{-3},\ldots ,\times A_{21}P^{-1},A_{22}P^{-2},\ldots \right)P^{+m}. \end{array}$$

After this transformation, the most important independent variable, $A_{11}$ , will no longer be a complex number but will become a positive real number, thus reducing the complexity of subsequent mathematical operations.

**Figure 2:**
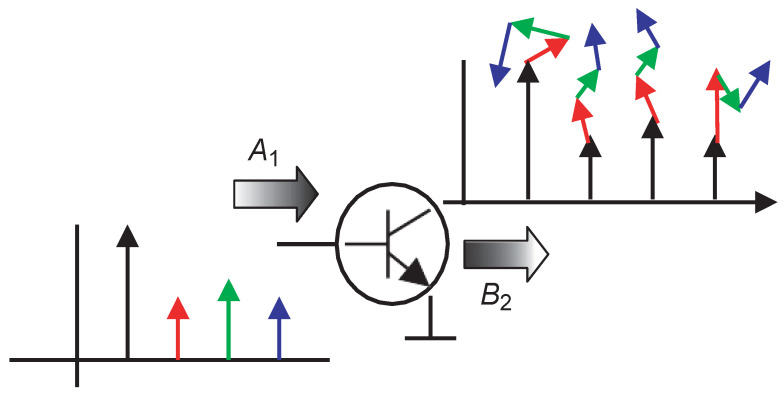
The harmonic superposition principle [11].

Assuming that, for a device-under-test (DUT), only the fundamental input signal, $A_{11}$ , is a large-signal excitation, and all other harmonic components are small-signal excitations, then the operating point of the DUT depends only on $A_{11}$ . Under this condition, the harmonic superposition theorem (as shown in Figure 2) can be applied to linearize these small harmonic signals. In this case, these small signals and their conjugate terms do not affect each other, and their effects on the output appear in a positive correlation form. Therefore, the final PHD model can be expressed as:

(4) $$\begin{array}{c} B_{pm}=\sum_{qn}\;S_{pq,mn}\left(\left|A_{11}\right|\right)P^{+m-n}A_{qn}+\sum_{qn}\;T_{pq,mn}\left(\left|A_{11}\right|\right)P^{+m+n}conj\left(A_{qn}\right). \end{array}$$

### 2.3. X-Parameter Model

In order to further promote and develop the model, Jan and Agilent jointly applied for a patent trademark, which is the X-parameter behavior model. Additionally, the description formula is written as:

(5) $$B_{ef}=X_{ef}^{\left(F\right)}\left(\left|A_{11}\right|\right)P^{f}+\sum_{gh\neq 11}\;X_{ef,gh}^{\left(S\right)}\left(\left|A_{11}\right|\right)A_{gh}P^{f-h}+\sum_{gh\neq 11}\;X_{ef,gh}^{\left(T\right)}\left(\left|A_{11}\right|\right)A_{gh}^{*}P^{f+h}.$$

**Figure 3:**
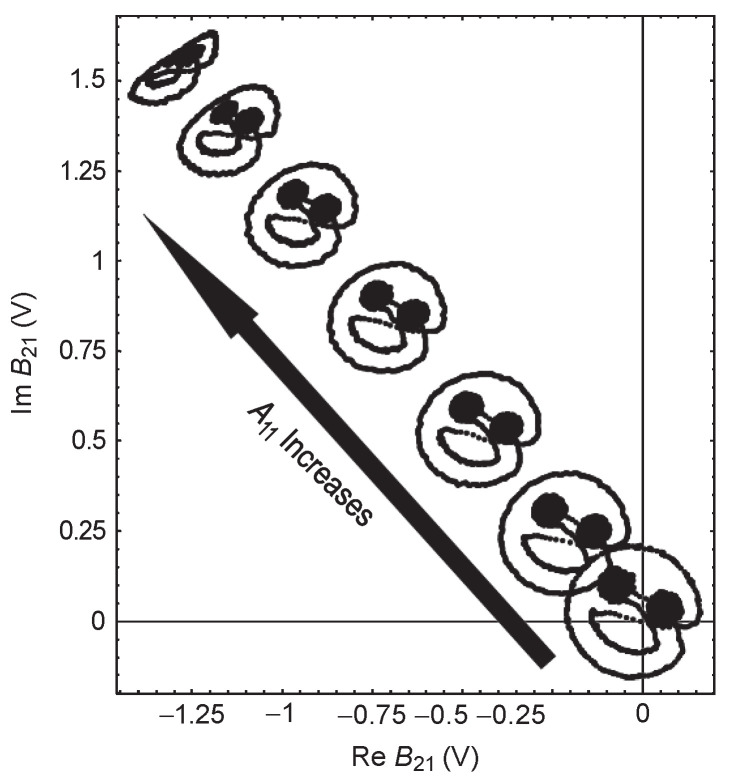
The conjugate term distorts the smiley face [11].

In Formula (5), the first item is determined solely by the large signal operating point. Only the large signal excitation is applied to the DUT, and then the amplitude and phase at all frequency points are recorded. By comparing these data with the amplitude and phase of the excitation signal, the amplitude ratio and phase difference of the cross frequency can be obtained to extract the parameters of this item. The $X_{S}$ and $X_{T}$ terms represent the influence of the perturbation small signal and the conjugate term of the perturbation small signal on the port scattering wave, respectively. (Although the reflection coefficient is not included in the model, the nonlinear response caused by the load impedance mismatch is actually included in the form of a small signal disturbance). Through Figure 3, it can be intuitively observed that, with the increase of $A_{11}$ , the nonlinear distortion of the system becomes more and more serious. The compression deformation of the smiley face is due to the existence of the $X_{T}$ term, and the rotation and scaling are affected by the other two.

According to the previous derivation process, the X-parameter’s large signal operating point depends solely on the nonlinear operating state of the device, including the input and DC bias information of the large signal, $A_{11}$ ( $|A_{11}|,DC$ ). Since the derivation is based on the single large signal excitation mentioned above, the model is primarily applicable to devices with good impedance matching. However, under conditions of significant load impedance mismatch, where the DUT operates in strong nonlinear conditions, the X-parameter model may exhibit a large prediction error.

### 2.4. Multitone Multiharmonic Scattering Parameters

In addition, References [12,13] proposed another method to extend the S-parameters, namely multi-tone, multi-harmonic S-parameters ( $M^{2}S$ ). The $M^{2}S$ parameters can be characterized by Formula (6) for each intermodulation product, and the spectral mapping relationship between *A* wave and *B* wave can be determined.

(6) $$B_{p,\left(k_{1},\ldots ,k_{N}\right)}\overset{\;\mathrm{def}\;}{=}S_{p,\left(k_{1},\ldots ,k_{N}\right)}\left[\left|A_{1}\right|,\ldots ,\left|A_{N}\right|\right]\prod_{n=1}^{N}\;\gamma_{k_{n}}\left(A_{n}^{|k_{n}|}\right).$$

Under the condition of selecting the appropriate asymmetric offset frequency, the model can avoid the overlap of intermodulation products, which can be characterized by independent $M^{2}S$ parameters, only depending on the amplitude $|A_{n}|$ and independent of the phase. Therefore, the $M^{2}S$ parameters can make it relatively simple to obtain a complete description. In fact, the characterization terms of the intermodulation products of different orders in the $M^{2}S$ parameters can correspond to the expansion of the Volterra series in the frequency domain and, according to its definition based on the description function, the $M^{2}S$ parameters can better model nonlinearity. When the two-port device is limited and the non-quasi-static memory effect is ignored, the $M^{2}S$ parameters can be extracted within the expected application specifications by selecting the appropriate offset frequency. At this time, the model can cover the entire working area of the DUT and has the ability to predict the behavior of the device under load-pull conditions. However, with the increase of nonlinear order, it is difficult to ensure that many intermodulation products do not overlap with each other, which limits the application of $M^{2}S$ parameters.

### 2.5. Load-Dependent X Parameter Model

As a nonlinear superset of S-parameters, X-parameters can be easily extracted by a nonlinear vector network analyzer (NVNA) [14,15]. However, the X-parameters measured separately only contain the waveforms of the harmonic and intermodulation product components under specified driving signal amplitude and frequency conditions. These measurements are typically confined to a 50-ohm characteristic impedance range. However, the optimal load impedance of the RF power transistor changes with the input power under large signal operating conditions. Therefore, it is necessary to be able to plot the equal output power or efficiency curve under different load impedance conditions and different input power on the Smith chart, in order to help designers find the best load impedance corresponding to the best performance point of the transistor [16]. This technique is commonly referred to as load-pull.

Combined with NVNA ’s measurement of X parameters and the load-pull measured method, X parameters can be obtained in a wide range of Smith charts [17]. The resulting behavior model is called the load-dependent X parameter [18,19], which is expressed as:

(7) $$\begin{array}{c} B_{e,f}=X_{of}^{\left(F\right)}\left(DC,\left|A_{11}\right|,\Gamma_{21}\right)P^{f}+\sum_{g,h}\;X_{f,ght}^{\left(S\right)}\left(DC,\left|A_{11}\right|,\Gamma_{21}\right)P^{f-h}\cdot A_{gh}+\sum_{g,h}\;X_{df,sh}^{\left(T\right)}\left(DC,\left|A_{11}\right|,\Gamma_{21}\right)P^{f+h}\cdot A_{gh}^{*}. \end{array}$$

**Figure 4:**
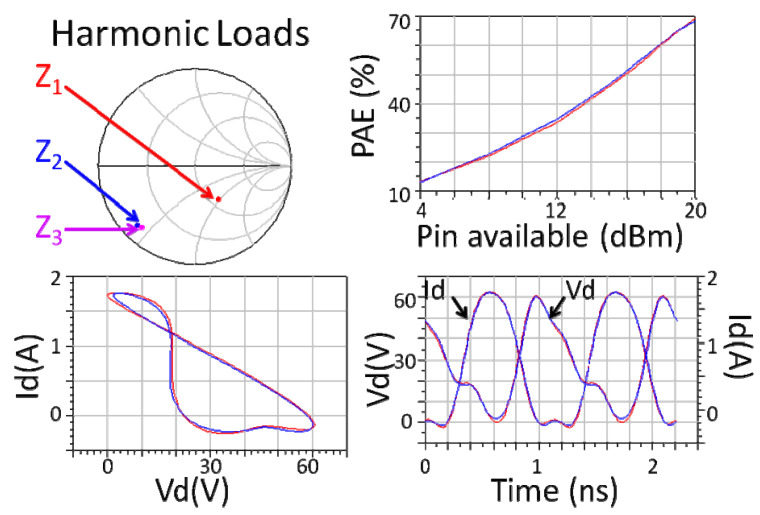
PAE (**upper right**), Id and Vd waveforms (**lower right**), and dynamic load-line (**lower left**) from load-dependent X-parameter model simulations (red) and measured time-domain harmonic load-pull validation (blue) at the harmonic impedances, $Z_{n}$ , specified in the (**upper left**) plot [19].

It can be seen from the expression of the behavior model that the so-called load correlation refers to controlling the value of the load reflection coefficient through the impedance tuner and returning part of the reflection signal of the large signal response on the device scattering port to the device as another large signal input to simulate the working conditions of the serious load mismatch. This model extends the validity range of X parameters. In essence, it collects X-parameter measurement data at different load impedance points and organizes them into a dataset so that designers can directly extract the corresponding X-parameter model for use in the region of interest, as shown in Figure 4. In fact, the method of directly using large signal test data, such as the load-related X-parameter model, is essentially a look-up table model [20].

However, because this model introduces more variables to define the large signal operating point, the size of the model file significantly increases, which limits the further use of the model.

### 2.6. Cardiff Model

In the meantime, the X-parameter model was developed, and a new modeling strategy was formed by combining the truth look-up table [21,22] with the polynomial behavior model. This strategy allows for the integration of measurement-based and model-based design methods, and enables the easy conversion of actual measured voltage and current waveforms into model parameters. This method can be easily integrated into CAD software and is called the Cardiff model. Furthermore, the Cardiff model also extends the classical X-parameter model, and its formula is expressed as:

(8) $$b_{k}=\sum_{m=0}^{\frac{n-1}{2}}\;C_{k,m}\cdot {\left(\frac{Q}{P}\right)}^{m}a_{1}+\sum_{m=0}^{\frac{n-1}{2}}\;U_{k,m}\cdot {\left(\frac{P}{Q}\right)}^{m}a_{2}.$$

Similar to the load-dependent X-parameter model, the Cardiff model also includes the amplitude $|A_{21}|$ of the incident wave at the output end, and separates the amplitude and phase of the large-signal incident waves into two different ports. The phase difference is represented by Q/P as an independent variable in the model function. *C* and *U* are the parameters of the model, which can be obtained by rotating and integrating the phase operators of different orders. This is a more general behavioral model, which can cover a large range on the Smith chart and expand the prediction range of the model by interpolation and extrapolation to cover the entire Smith chart [23,24].

However, since the model does not define the high-order harmonics of the reflected wave, it is only suitable for simulations related to the fundamental wave output, such as fundamental wave load-pull or AM-AM, AM-PM distortion. In the strong nonlinear region, the prediction error may increase. Therefore, we consider extending the original single-input Cardiff model to two-tone or multi-tone inputs to model mixed-order and intermodulation products [25]. It can also be used to predict the behavior of a multi-input single-output amplifier (MISO PA) [26]. In this model, there are at least two interacting excitation signals with different amplitudes and phases, which can be expressed as:

(9) $$\begin{array}{c} B_{p,h}={\left(∠A_{1,1}\right)}^{h}\cdot \sum_{x}\;\sum_{m}\;\sum_{n}\;\ldots ,K_{p,h,m,n,x}\cdot {\left|A_{1,1}\right|}^{x}\cdot {\left|A_{2,1}\right|}^{m}\cdot {\left(∠\frac{A_{2,1}}{A_{1,1}}\right)}^{n}. \end{array}$$

In this model, the subscripts x and m represent the amplitude variation range of the two signals, and n represents the phase variation range. The model normalizes the phase to the fundamental phase of $A_{1,1}$ to ensure that each harmonic is aligned when the fundamental phase is 0°. Similarly, the model coefficients are separated into a form that is only related to the amplitude and is independent of the phase, which simplifies the mathematical expression and maintains the time invariance of the model. Through verification experiments, it is proven that this model has excellent interpolation ability and greatly reduces the original dataset that needs to be used for modeling while maintaining good prediction accuracy. However, due to the need to re-normalize the traveling wave, the complexity of model extraction is high. In addition, in the [27], by incorporating DC bias into the formula of the Cardiff model, the versatility of the model is further improved and the test intensity is reduced.

### 2.7. Dynamic X-Parameters Model

As the power of the power transistor excitation signal is further improved, the signal with a high peak-to-average power ratio will drive the transistor to a fully saturated state, making it work under strong nonlinear conditions and exhibit a memory effect [28,29]. The existence of the memory effect means that the instantaneous output of the device at any time depends not only on the instantaneous input at the current time, but also on the input of the device at all past times. This makes the previous model unable to accurately predict the output response of the device [30].

In order to further extend the X parameter to represent the existence of long-term memory effect in the envelope domain, a model called dynamic X parameter is proposed [31,32]. The equation is:

(10) $$\begin{array}{c} B\left(t\right)=\left(F_{CW}\left(\left|A(t)\right|\right)+\left.\int_{0}\;\right.G\left(\left|A(t)\right|,\left|A\left(t-u\right)\right|,u\right)du\right)\exp\left(j\varphi \left(A(t)\right)\right). \end{array}$$

The basic idea of this model is to combine a static function $F_{CW}\left(\cdot \right)$ describing nonlinear behavior with a dynamic nonlinear function G(·) over the time integral function to characterize the output. The static part here can be regarded as the classical PHD model, which represents the large signal envelope domain response of the device under the excitation of a given input signal. The dynamic part is the integral of the general nonlinear function of the instantaneous amplitude of the input signal A(t) , the past value of the input signal A(t−u) , and the time when the past value occurs (variable *u*). This also explains the correlation between the instantaneous response of the device and the current input amplitude, the past input amplitude, and the time span between the two in the memory effect. Therefore, the extended dynamic X-parameter behavior model has been proved to be able to predict the strong nonlinear behavior and the memory effect caused by a wider range of input signals.

In general, the above models perform well under certain conditions but, considering the diversity and complexity of RF devices, no model can fully cover all working conditions. The selection of appropriate models should be based on specific application scenarios and device characteristics of concern and, as the components to be characterized continue to increase, the complexity of the model and the difficulty of extraction also increase. Future research may require more comprehensive and general models to more accurately describe the nonlinear behavior of various RF devices. This may involve integrating the advantages of different models to create a more comprehensive and adaptable behavior model.

### 2.8. Other Numerical Models

In addition to the series of models described above, a large number of behavior models based on numerical calculations have emerged in the study of the nonlinear behavior of RF power devices [33,34,35]. These models can be roughly divided into two categories [36].

The first type is a memoryless model suitable for early communication systems. For communication systems with narrow bandwidth, the distortion generated by the signal is generally static nonlinear distortion, that is, the output of the system only depends on the input signal at the current time. This kind of model can be fitted by a power series model and the Saleh model [37,38].

The second type is a memory nonlinear model for modern broadband systems, which is originally developed based on the Volterra series model [39,40,41]. The Volterra series is a generalization of the Taylor series, which integrates the memory term into the Taylor series [42]. However, with the increase in memory depth and nonlinear order, the number of Volterra series kernels will increase significantly, making it difficult to identify the parameters of the model. Therefore, this series of models is mainly used to describe weakly nonlinear systems.

In addition, the proposed piecewise linear function model has formed another new route [43]. This method uses linear functions to fit nonlinear behaviors in each interval and smooth transition functions between different segments to ensure continuity. Based on this idea, Cai et al. [44] proposed a model based on a multidimensional canonical section-wise piecewise linear (CSWPL) function. The traditional CPL method only supports univariate description functions and can be extended to model complex signals. The model structure is the same as the Cardiff model, and the multi-dimensional CSWPL is used for an approximate description. The multi-dimensional variables used to extract the model parameters come from the frequency domain data and the DC component under different input power and load impedances obtained by load-pull measurement, and then the parameters are solved by the least square method. The established model can cover the entire Smith chart with a set of fixed parameters, and is compared with the artificial neural network (ANN) model, the polynomial model, and the PHD model. Only the ANN model can have a similar prediction accuracy to the CSWPL model in areas with strong nonlinearity, but the complexity of the ANN model is much higher than that of the CSWPL model. Therefore, the CSWPL model has become a flexible and efficient behavioral model with excellent performance in highly nonlinear modeling.

## 3. Machine Learning Based Model

**Figure 5:**
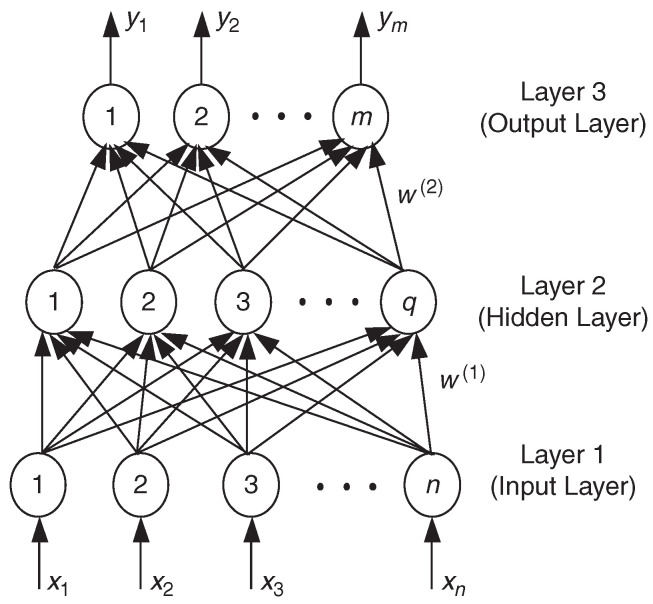
A three-layer perception neural network structure with an input layer, a hidden layer, and an output layer. Generally, a multi layer perception network consists of an input layer, one or more hidden layers, and an output layer [47].

The neural network is proposed by imitating the working principle of the human brain [45]. It performs correlation operations by constructing artificial neurons. The artificial neuron is composed of neurons, weights, summation units, thresholds, and activation functions, as shown in Figure 5. The structure and weight of the neural network will be adjusted according to the change of the external input signal. By continuously learning and training the sample data provided, the neural network can find the relationship between the input and output signals to construct the model [46].

Since the 1990s, machine learning has gradually entered the field of RF CAD in the form of ANN [48]. ANN learns the behavior of RF and microwave devices and circuits through training, combines the knowledge of RF devices with ANN, and uses machine learning methods to improve existing behavioral models and even create new models [49,50,51,52,53,54].

The application of neural networks in RF device behavior modeling is similar to the idea of polynomial fitting modeling, although such models are not as accurate as physical models. In recent years, the development of neural networks has made great progress, and networks with different structures and training algorithms have emerged in an endless stream. Therefore, the application of these diverse neural network structures to establish the nonlinear behavior model of power devices has gradually received attention.

### 3.1. Time-Delay Feedforward Neural Network Model

In the initial stage of the study, the behavior model based on a multi-layer perceptron (MLP) network has emerged, which has a simple structure and can model the behavior of a memory-less RFPA. However, as mentioned above, the memory effect is unavoidable. In order to solve this problem, a time-delay feed-forward neural network model is proposed in this paper [55]. The input layer of the model contains the time-domain voltage data and its delay response term, and the output layer is the sample collected in the time domain by testing. This neural network constructs a form of expression that conforms to the time-domain memory effect, that is, the output of the device depends not only on the input at the current moment, but also on the state at other moments. By selecting the appropriate input delay, the model can fully represent the memory effect of the model. By providing multiple sets of training data with different input, output, and delay, the model can obtain a wider representation ability.

Additionally, some models incorporate the training method of a feedforward neural network with a global recurrent network. This hybrid approach captures substantial dynamic information, contributing to the convergence of a high-precision model [56,57].

### 3.2. Dynamic Neural Network Model Series

In order to make the neural network an accurate model of the problem to be learned, it is necessary to set the number of neurons and the number of hidden layers reasonably, which depends on the degree and order of the nonlinearity of the device. Complex nonlinear behavior requires more neurons. In general, users can adjust the hidden layer through experience or trial and error, but this is a costly way. Therefore, the dynamic neural network that recruits and prunes neuron nodes through learning algorithms has become a new choice [58].

**Figure 6:**
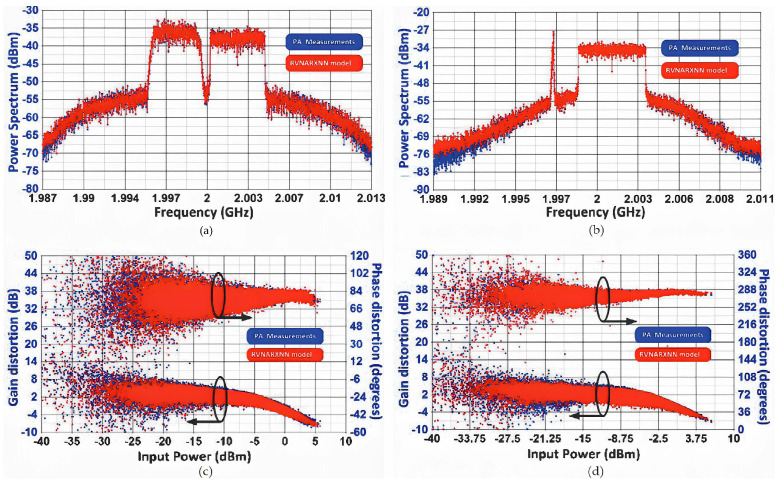
(**left**) Comparison between the RVNARXNN behavioral model and the measured data of the WCDMA of the 5 MHZ and the LTE of the 5 MHz signal. (**right**) Comparison between the RVNARXNN behavioral model and the measured data of the GSM and the LTE of the 5 MHz signal. (**a**,**b**) Output power spectrum. (**c**,**d**) AM–AM and AM–PM characteristics [59].

In the paper [59], a model based on a nonlinear autoregressive external input neural network (NARXNN) is proposed. The model adds two branch delay lines and a feedback mechanism, which can memorize and process historical data. The number of input/output layer nodes is set according to the number of input/output values. It is a dynamic neural network, which has advantages in time-related sequence prediction including the PA memory effect. Using this model, a good prediction effect can be obtained. As shown in Figure 6, the model is highly correlated with the measured results.

In contrast, of neural network models with large parameters, typical examples include the GPT (Generative Pre-trained Transformer) series models developed by OpenAI. These models are known for their powerful learning and representation capabilities, and are capable of transfer learning on specific tasks through pre-training trials. However, there is no research on the application of artificial intelligence based on large models to the modeling of the nonlinear behavior of RF circuits, and we will continue to pay attention to the research progress related to this direction.

### 3.3. Support Vector Regression (SVR) Technology Series

However, the neural network-based model has inherent overfitting problems and its generalization ability is poor. In addition, the ANN model usually cannot determine the optimal model structure, so the modeling efficiency is not high. Therefore, more and more people begin to use SVR technology based on the core of the machine learning algorithm to model behavior [60]. Compared with the ANN model, SVR technology is more efficient and stable. The description function of the SVR model is defined as follows:

(11) $$B_{pm}=f_{pm}\left(\overset{︷}{A_{qn}}\right)=\sum_{i=0}^{k}\;\beta_{pm,i}K\left(\overset{︷}{A_{qn,i}},\overset{︷}{A_{qn}}\right)+b_{pm}.$$

**Figure 7:**
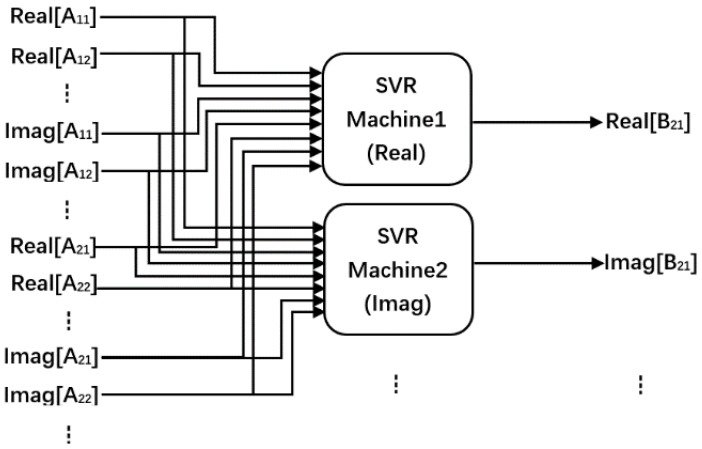
Block diagram of the SVR based model [61].

According to the block diagram of the SVR model shown in Figure 7, it can be seen that the model separates the real and imaginary parts of the input signal. It then inputs them into two SVR machines, respectively, and the two SVR machines output the real and imaginary parts of the reflected signal, respectively. The SVR model can effectively predict the behavior of the device at the fundamental and higher harmonic frequencies in the Smith chart range.

In the follow-up study, Ref. [62] proposed the LS-SVR technology and optimized the SVR model to provide a more efficient model extraction process without reducing the accuracy of the model.

## 4. Measurement Techniques and Model Optimization

The key to obtaining a high-precision behavior model is the quality of test data, and obtaining high-quality test data requires the support of advanced and reliable measurement technology. In today’s highly interconnected communication and RF fields, based on the demand for higher data rates and a wider spectrum, traditional linear measurement methods can no longer meet the requirements for in-depth understanding and accurate evaluation of dynamic system trend changes, time-varying relationships, and interactions between parameters. Therefore, the importance of nonlinear measuring instruments and technology is particularly significant. Especially for the behavioral model, it cannot be scaled by simple parameter changes after the modeling is completed, as the physical model does, and it relies more on accurate and efficient measurement techniques. This section will discuss the continuous expansion of vector network analyzers, show how the evolution of measurement technology promotes the development of behavioral models, and introduce the impact of model optimization.

### 4.1. Vector Network Analyzer (VNA)

VNA is one of the earliest test tools used in the RF field. It measures the response of the network as a vector, including real and imaginary parameters, in order to facilitate the capture of signal amplitude and phase. Its main working mode is to measure the transmission, reflection, impedance parameters, and S parameters in the frequency band of interest to analyze the performance of various circuits and devices.

**Figure 8:**
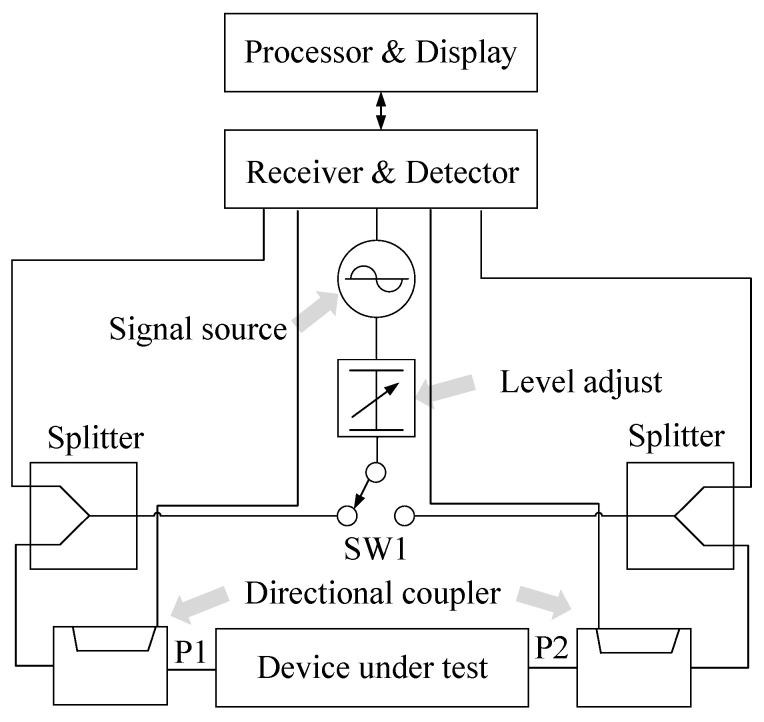
The structure of the VNA.

The structure of the VNA is shown in Figure 8, where the signal source is used to generate the excitation, which is transmitted to different directions of the DUT according to the test requirements through the splitter. The input signal and output signal on the DUT are separated by the directional coupler of the two ports, and then the RF signal is measured and processed in the receiver part to obtain the required frequency, amplitude, phase, and other information.

### 4.2. Large Signal Network Analyzer (LSNA)/Microwave Transmission Analyzer (MTA)

**Figure 9:**
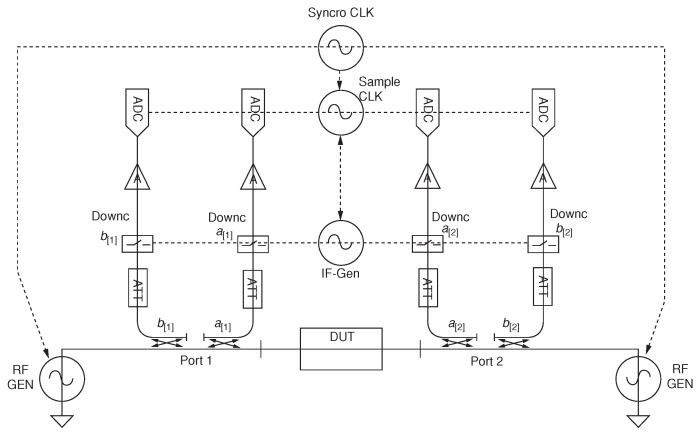
Simplified block schematic of a two-port LSNA [63].

The traditional VNA measurement has nothing to do with power, which makes it limited to the description of the LTI system. In order to overcome its limitations under nonlinear conditions, LSNA and MTA gradually emerged. LSNA obtains the absolute amplitude of the wave and the absolute value of the phase relationship between the harmonics in the complete range by performing fast Fourier transform (FFT) on the entire spectrum. Its structure is shown in Figure 9.

Similar to VNA, LSNA uses RF signal generators and couplers to transmit excitation signals and acquire incident and reflected waves. In the subsequent processing of the captured RF signal, an attenuator is needed to avoid the subsequent components working in the nonlinear region. Due to the high frequency of the signal, it cannot be directly digitized, so it is necessary to perform down-conversion. This part of the device is actually composed of MTA and is realized by the sampling theorem. LSNA also uses a 10 MHz synchronization line to synchronize the clocks of various components of the instrument to obtain an accurate coherence relationship between phases.

**Figure 10:**
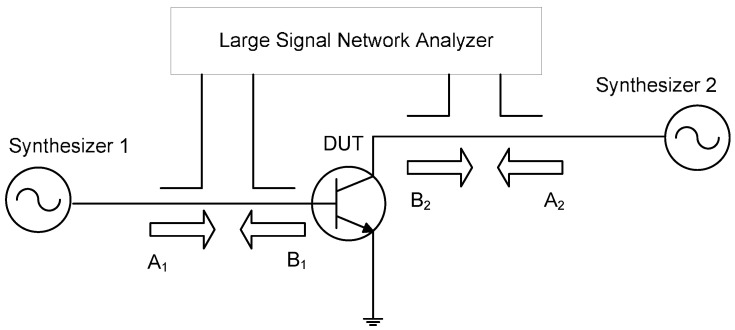
Distortion hot S-parameters measurement setup [65].

For nonlinear devices that do not consider the memory effect, LSNA can already obtain more reliable test data. For example, the Hot-S parameters can be extracted by the test system [64] shown in Figure 10. The system structure is very simple, consisting of a large signal network analyzer and two signal generators. Among them, signal source 1 provides a large signal ( $A_{1}$ ) that puts the system in a ‘hot’ condition, while signal source 2 generates a small signal ( $A_{2}$ ) independent of ( $A_{1}$ ). The test process is similar to the traditional S parameter measurement. The measured data can be substituted into Formula (2) to obtain the Hot-S parameter.

For such a simple test system, it mainly depends on the powerful instrument of LSNA. However, as the complexity of RF and microwave technologies increases and test requirements become more diverse, combining network analyzers with other devices is an effective way to scale.

### 4.3. Nonlinear Vector Network Analyzer (NVNA)

**Figure 11:**
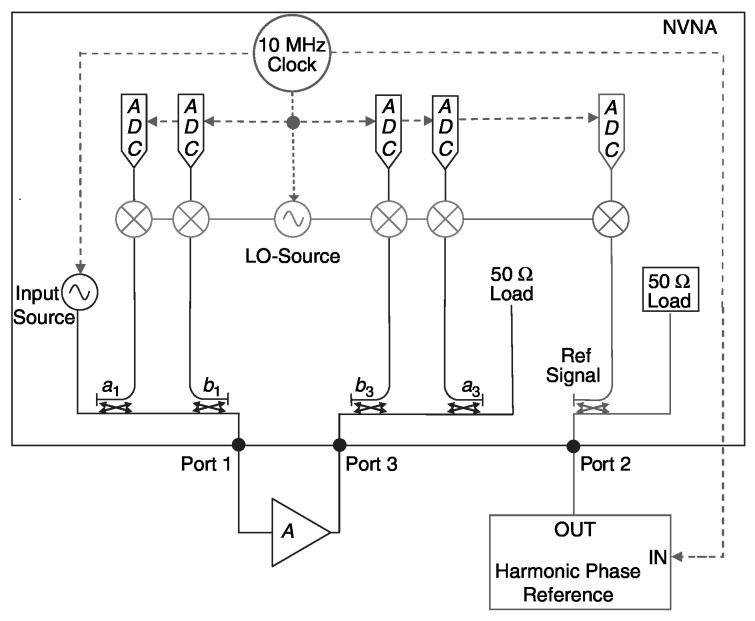
Simplified block schematic of a nonlinear vector network analyzer (LO represents the local oscillator signal) [66].

Since the LSNA measures the entire spectrum at the same time, the grid space for phase calibration will be larger if the interpolation algorithm is not used. NVNA achieves similar functions to LSNA in another way, and its structure is shown in Figure 11.

Unlike LSNA, NVNA is based on the heterodyne principle and uses a mixer to convert the RF signal into an intermediate frequency. Since NVNA only measures one frequency component at a time, all the spectral lines are correctly spliced together to obtain a complete measurement result, which requires the harmonic phase reference to be controlled by the synchronous clock signal in order to finally restore the relative phase relationship between the harmonic components. Based on NVNA, the X-parameter hardware test platform can be constructed, and the load impedance can be changed by controlling the impedance tuner to realize the measurement of load-pull data [18].

### 4.4. Pulse Measurement Technology

Different from the electrical memory effect mentioned above, when the instantaneous power of the PA fluctuates with the change of the input signal, the junction temperature of the transistor changes, which leads to the thermal memory effect. This effect is mainly manifested as the self-heating and trap effects, resulting in the decrease of PA efficiency, output power, and linearity. In order to reduce the influence of device self-heating on the measurement results and to better analyze the trap effect, pulse measurement technology is a reasonable choice.

#### 4.4.1. Pulse-IV Technology

For general transistors, IV characteristics can be characterized by applying a short-time low-duty-cycle pulse signal. However, the asymmetry of charge trapping and recovery time in GaN FET requires new measurement methods for accurate characterization. Ref. [67] proposed a dual-pulse measurement technique. The technique uses two pulses, one of which puts the transistor in a preset trap state, while the other provides an actual IV measurement. Through this pretreatment method, a customized dual-pulse IV of PA under different operating conditions can be obtained to predict more achievable PA performance.

#### 4.4.2. Pulse-RF Technology

**Figure 12:**
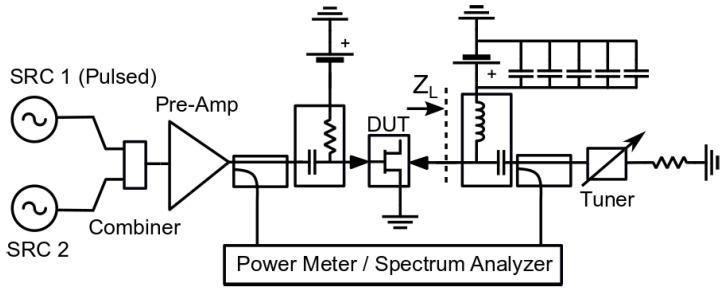
Block diagram of the measurement setup [68].

After the pulse IV measurement of the transistor, the characteristic information of its knee point drift and current collapse can be obtained. However, the transient effect of the change of the working point of the gauge on the RF performance of the device cannot be obtained. In order to overcome the limitation of static measurement and obtain the response in a more application-like environment, it is necessary to perform pulse RF large signal measurement. For example, Reference [68] proposes to evaluate the nonlinear dynamic effects by measuring the transient behavior under pulsed RF excitation. In general, IM3 is widely used to characterize the memory effect. However, when considering the trap effect, it is only related to the envelope frequency below 1MHz, so it must be measured by a very small pitch interval, which will inevitably lead to a decrease in measurement accuracy. The measurement system shown in Figure 12 is used for data acquisition.

The system can simulate different self-heating and trap conditions by changing the pulse power and duty cycle, so this measurement method can effectively characterize the transient behavior of devices with trap effects.

By comprehensively applying these pulse measurement methods, the basic IV characteristics of the transistor can be obtained while obtaining transient characteristics that are in line with the actual application conditions. Just as in Ref. [69], for devices with different Fe-Doped buffer concentrations, there is no significant difference in the characteristics obtained by traditional measurements such as pulse IV measurement. It can be supplemented by a pulsed RF measurement, and the effect of doping concentration on the recovery time after the current trap is found. The development of such pulse measurement techniques provides more favorable data support for the establishment of models that can characterize long-term memory effects [70,71,72].

### 4.5. Load-Pull Technique

**Figure 13:**
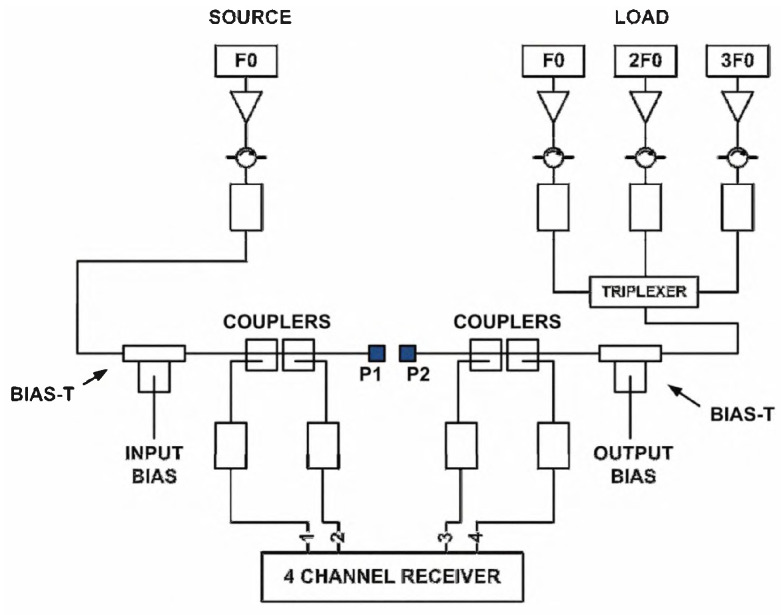
Active load-pull measurement system [73].

The influence of load-pull data on the accuracy of the model cannot be underestimated. In addition to the passive load-pull realized by the impedance tuner in the above test system, an active load-pull measurement system is proposed in Figure 13.

The main difference is that the signal generator with the same phase reference is used to actively inject the signal at the output end to form the required reflection coefficient. After passing through the tripler, it enters a 4-channel receiver to change the load impedance of the DUT, in order to achieve the effect of load-pull. This measurement method can also be used to extract the parameters of the harmonic Cardiff model, which can be used to model the device under strong nonlinear states.

**Figure 14:**
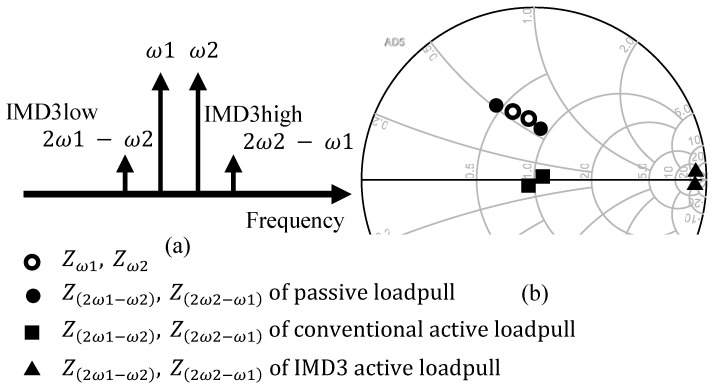
IMD3 load-pull measurement: (**a**) frequency domain; (**b**) impedances on a Smith chart [77].

However, with the enhancement of the nonlinearity of the device, the memory effect cannot be ignored. In this case, it is difficult to accurately measure high-order nonlinear behaviors (such as intermodulation distortion) only through a single-tone test system, and relevant information can only be obtained through a two-tone test or even a multi-tone test [74,75,76]. These existing test techniques cannot fully describe the nonlinear components in the memory effect. With the advent of the 5G era, the amount of data in the communication system is increasing, and the bandwidth and frequency are also higher. Therefore, it is a foreseeable development trend to further combine the two-tone, non-equidistant multi-tone measurement with load-pull technology, which will be a test technology closer to reality. For example, in the article [77], the impedances of the fundamental frequency and IMD3 are controlled by using different VSGs, as shown in Figure 14, so that they can cover the entire Smith chart. This measurement method can effectively determine the true application potential of the transistor, thereby optimizing the performance of the final design product.

### 4.6. Model Optimization

In addition to the development of nonlinear measurement technology, more and more research is currently being devoted to optimizing behavioral models. By optimizing the model parameters and structure, the fit and performance of the model can be improved to make it closer to the actual observation results. Additionally, the model extraction algorithm can be improved to ensure faster convergence speed and higher reliability without sacrificing representation accuracy.

In the traditional Cardiff model coefficient extraction method, the linear least squares method is usually used for calculation. However, this mathematical calculation process can become complicated when dealing with a large amount of data. In the article [50], a new method is proposed to optimize the original model by using artificial neural networks to extract the coefficients of the Cardiff model. Different from other neural networks, this method divides the input and output data of the device into real part and imaginary part matrices, and inputs them into the neural network simultaneously. Moreover, the expected model coefficients are used as the target output of the output layer and are then trained. By using this method to extract the model, acceptable accuracy can be achieved while simplifying the calculation process, thus optimizing the model.

This method not only simplifies the calculation process of Cardiff model coefficient extraction, but also improves the efficiency and performance of the model. This method of optimizing the model represents important progress in the field of behavioral models and is expected to yield better results in practical applications.

In Ref. [78], a method for extracting models from large-signal measurement data is proposed. This method generates a model function of any density by introducing a new nonlinear function sampling (NFS) operator (similar methods are also used in the $M^{2}S$ parameter extraction process). Based on the linear dependence on the unknown quantity, the extraction process is completed by solving the global linear equations of the unknown spectral components of the charge waveform, and finally the automatic model parameter identification is realized. This is also a major development direction of model optimization.

## 5. Applications

In the field of modern RF and microwave, nonlinear behavior models have evolved into indispensable tools. These models are not only used to describe the performance characteristics of devices/circuits, but also enable in-depth understanding of electronic systems and convenient simulation to meet growing demand, reduce design costs, improve performance, and promote innovation. In the face of the increasing complexity of electronic system design and optimization, the behavior model of RF power devices presents an exciting application prospect.

### 5.1. Design and Optimization of RF Power Amplifier

The requirements of modern communication systems for power and efficiency are increasing day by day. As the core component of RF links, the nonlinear behavior of power amplifiers has a significant impact on the overall performance. Especially in complex signal modulation and high input power scenarios, the nonlinear characteristics of PA may lead to signal distortion, spectrum proliferation, and unnecessary electromagnetic interference. Therefore, the establishment of a highly accurate transistor model becomes a prerequisite for optimizing PA design and developing linearization techniques.

In [79,80,81,82,83], behavioral models have been used to analyze various distortion mechanisms such as device intermodulation and harmonic distortion in detail, and began to explore PA linearization techniques such as predistortion and feedback. For example, in [84], T. S. Nielsen et al. extracted an X-parameter model of the Gan HEMT power transistor by using the nonlinear vector network analyzer NVNA and, based on this model, the design and development of Doherty PA was completed. In [85], a behavior model-based algorithm is proposed to increase the speed and accuracy of multi-tone wideband load-pull. Finally, after actual measurement and verification, it was found that the deviation between the test results and the simulation remained at a relatively low level, which also proved the effectiveness of the behavior model.

### 5.2. Signal Integrity

In today’s high-frequency communication environment, it is very important to ensure the transmission quality and accuracy of high-frequency signals from the transmitter to the receiver. Especially in the complex electromagnetic environment, the influence of various interferences and distortions makes the signal transmission more vulnerable, which may lead to data loss, timing errors, and overall performance degradation. The traditional electromagnetic modeling method based on Maxwell’s equations may be limited in complex processes. Therefore, nonlinear behavior modeling of transmission circuits has become an innovative solution to the problem of signal integrity in high-speed circuit design.

For working conditions with complex interference, such as electromagnetic radiation, electromagnetic induction, and electromagnetic scattering, the traditional model may find it difficult to fully consider the influence of nonlinear behavior. Therefore, behavior models based on machine learning, such as CNNs, have become a more intelligent and efficient solution. Through the study of a large number of actual data, these models can capture the complex behavior in the nonlinear system, enabling them to predict key signal quality indicators more accurately and provide a more accurate reference to improve the fault tolerance and anti-interference ability of the signal.

### 5.3. Electromagnetic Interference Field

As the electromagnetic environment becomes more and more complex, electronic equipment and systems are facing increasingly serious electromagnetic compatibility challenges. The existence of electromagnetic interference may lead to communication interruption, equipment failure, and even potential risks to personal safety. Therefore, the modeling and simulation of electromagnetic interference have become key steps in preventing, identifying and solving electromagnetic compatibility problems in advance, which is another important application field of nonlinear behavior models. RF devices operating in extreme radiation environments must be able to withstand high radiation levels and maintain their performance degradation within an acceptable range. Therefore, the modeling of devices under severe radiation has become an attractive field. Some of the early models were extended on the basis of the SPICE model, considering the effects of gamma rays, neutron radiation, etc. [86,87], but there was no clear extraction process of radiation parameters, and it was not convenient to use. Subsequently, in [88], the researchers established proton and neutron damage models with the help of TCAD, and achieved a good agreement with the measured results. In addition, Sichen Yang et al. [89] realized a model with certain nonlinear parameter prediction ability for the RSE problem by combining a series of methods mentioned above.

In [90], through a large signal network analysis of nonlinear systems, the extracted X parameters are used to measure the immunity of the system under electromagnetic interference. The results show that the behavior model has wide application potential in electromagnetic compatibility modeling and simulation. This application potential is not only reflected in theoretical research, but also has substantial value in system design and performance optimization in the actual electromagnetic environment.

In short, the nonlinear behavior model has a wide application prospect in the field of RF and microwave, which is helpful in improving the performance of electronic systems, promoting technological innovation, and ensuring the normal operation of electronic equipment in a complex electromagnetic environment. These behavioral models play an indispensable role in modern communication, computing, and electronic systems, helping engineers solve evolving technical challenges by improving the accuracy and intelligence of the models. Therefore, the application of the nonlinear behavior model is not only the demand of the current RF microwave field, but also the guidance for the future development of electronic systems. By continuously improving and expanding these behavioral models, we can expect to see more innovative products and solutions to meet the ever-increasing performance and complexity requirements. In engineering practice, these models will continue to play a key role in promoting the continuous progress of RF microwave technology.

## 6. Challenge and Prospect

As a key research direction in the electronics field, the modeling of RF front-end circuits and devices continues to show a wide range of prospects through the support of innovative measurement techniques, model optimization methods, and diverse application fields. This paper discusses the advantages and disadvantages of the behavioral model as a modeling tool, emphasizing its lack of a clear physical basis, the limitations of geometric scaling expansion, the problem of high dependence on data quality and quantity, and the challenge of re-fitting when working conditions change. In this context, this paper proposes the potential prospects of hybrid models, and believes that the combination of physical models and behavioral models is the future development direction.

First of all, the advantage of the hybrid model is that it can introduce physical knowledge to guide the model construction and constrain the parameter extraction process, thereby improving the interpretability of the model. The combination of data fitting of the behavior model and the intuitive reflection of the physical model on the device behavior can not only improve the accuracy and generalization ability of the model, but also reduce the high dependence on a large number of test data, thereby reducing the measurement cost and time. Based on the support of physical knowledge, the parameters in the behavior model can also contain a certain physical meaning, so that the hybrid model has a certain geometric scaling ability and can be extended to other working conditions. Therefore, the hybrid model has become an effective way to deal with practical engineering problems.

At the same time, it provides a variety of model selection guidance for different practical application scenarios. Especially in the modeling of complex nonlinear behavior, the model based on machine learning highlights the new direction and method for RF power device modeling research. Through this application, the behavior of the device can be simulated and analyzed more accurately, providing more possibilities for RF circuit design.

In addition, this paper also emphasizes the key role of measurement technology. The traditional VNA provides a basis for us, but with the continuous upgrading of RF system requirements, the introduction of new measuring instruments such as LSNA, MTA, and NVNA makes it possible to understand nonlinear systems in depth. Load-pull technology provides a more complex and comprehensive test method under different impedance conditions by introducing active and passive means. Subsequent measurement techniques can be further developed to include complete measurements of device memory effects. At the same time, the model optimization method also provides a new idea for improving the accuracy and efficiency of the model. The comprehensive application of these advanced measurement techniques and model optimization methods will inject more vitality and possibility into the future development of RF power device behavior models.

In short, RF power device modeling is a promising field, not only because of its wide application in electronic system design and optimization, but also because of its innovative research directions and methods. Future research will continue to explore how to better integrate various models to meet the growing demand for RF applications, while emphasizing the importance of data quality, model accuracy, and interpretability to ensure that behavioral models give full play to their potential in practical applications. Hybrid models, neural network models, advanced measurement techniques, and model optimization methods will jointly promote the future development of RF power device modeling.

## Data Availability

Not applicable.
